# Comparative outcomes of robotic, laparoscopic, and open hysterectomy for complex hysterectomy: a retrospective study

**DOI:** 10.3389/fsurg.2025.1739304

**Published:** 2026-01-27

**Authors:** Bowei Li, Shaohan Ma, Zhuo Xiong, Xiuxin Chen, Xinyi Qi, Yuan Ma, Ruyue Li, Ruigi Zhang, Xinshu Li, Chunfang Ha

**Affiliations:** 1 The First Clinical Medical College of Ningxia Medical University, Yinchuan, Ningxia, China; 2 Yinchuan Maternal and Child Health Care Hospital, Yinchuan, Ningxia, China; 3 Department of Gynecology, General Hospital of Ningxia Medical University, Yinchuan, Ningxia, China

**Keywords:** complex large uterus, Da Vinci robot surgery, hysterectomy, laparoscopic surgery, laparotomy surgery

## Abstract

**Background:**

The optimal surgical approach for hysterectomy in patients with complex large uteri remains controversial, particularly in those with uterine enlargement and obesity. This study aimed to compare perioperative outcomes and complication rates among three surgical techniques—open abdominal hysterectomy (LH), traditional laparoscopic hysterectomy (TLH), and Da Vinci robot-assisted hysterectomy (DV-RH)—to provide evidence for individualized surgical decision-making.

**Methods:**

A retrospective analysis was conducted on 444 patients who underwent hysterectomy for benign gynecological diseases between April 2021 and April 2024 at the Ningxia Medical University General Hospital.Patients were divided into LH (*n* = 140), TLH (*n* = 179), and DV-RH (*n* = 125) groups. Baseline, perioperative indicators, and the incidence of both short- and long-term complications among the three groups were compared. Additionally, BMI was stratified to compare the related indicators separately.

**Results:**

There were no significant differences in age, BMI, comorbidities, or surgical history among the groups. DV-RH showed significantly lower blood loss (*P* < 0.001), postoperative VAS scores (*P* < 0.001), and complication rates, especially in patients with uterine size ≥16 gestational weeks. TLH demonstrated comparable outcomes to DV-RH in selected indicators but had a higher incidence of Clavien-Dindo grade II complications (*P* = 0.032). Hospitalization costs were highest in the DV-RH group (*P* < 0.001).

**Conclusion:**

DV-RH offers superior perioperative outcomes in complex and large uterus cases but incurs higher costs. TLH remains a cost-effective alternative in appropriate patients. Surgical decisions should be based on patient characteristics, technical complexity, and institutional resources.

## 1 Introduction

Hysterectomy represents one of the most common surgical procedures in gynecology, primarily indicated for benign uterine conditions such as uterine fibroids and adenomyosis (1). These disorders frequently lead to enlargement of the uterus, distortion of pelvic anatomy, and, in many cases, coexisting pelvic adhesions (1). In clinical practice, patients with a voluminous uterus—typically defined as equivalent to ≥12 weeks of gestation in size—and abnormal uterine morphology are classified as having a complex large uterus. Surgical management of such cases presents significant technical challenges due to restricted pelvic space, increased vascularity, and limited exposure. Adding to this complexity is the rising prevalence of obesity among women of reproductive and perimenopausal age, a factor that independently increases surgical risk. Obesity has been associated with higher intraoperative blood loss, longer operative times, and increased rates of postoperative complications, particularly wound infections and thromboembolic events (2, 3). These risks are further amplified when treating obese patients with a significantly enlarged uterus, underscoring the need for surgical techniques that offer both precision and minimal invasiveness. As a result, the choice of surgical approach plays a pivotal role in minimizing perioperative risks and enhancing recovery outcomes.

Currently, three primary surgical techniques are widely adopted for hysterectomy in patients with complex large uteri: Open abdominal hysterectomy (laparotomy, LH), Traditional laparoscopic hysterectomy (TLH), and Da Vinci robot-assisted laparoscopic hysterectomy (DV-RH) (4, 5). Each approach has its own set of advantages and limitations. Open hysterectomy, while offering direct visualization and tactile feedback, is associated with larger incisions, increased postoperative pain, longer recovery times, and higher risk of wound-related complications (2). Traditional laparoscopy offers well-established benefits in reducing hospital stay and surgical trauma (1); however, its efficacy may be compromised in large uteri or in obese patients due to poor visualization, reduced instrument mobility, and increased conversion rates to laparotomy (6, 7). In contrast, robotic-assisted laparoscopic surgery—particularly using the Da Vinci surgical platform—has emerged as a valuable option for complex hysterectomy cases. The system's enhanced dexterity, three-dimensional visualization, and tremor filtration facilitate precise dissection and suturing in anatomically constrained spaces (3, 8). Studies have demonstrated its potential in reducing intraoperative blood loss, postoperative pain, and complication rates, particularly in patients with obesity or large uteri (9). Nonetheless, the widespread use of robotic-assisted surgery is constrained by its high cost, limited availability, and steep learning curve.

Despite these advancements, clinical consensus on the optimal surgical route for hysterectomy in patients with large uteri and/or obesity remains lacking. Most existing studies focus on average-sized uteri or non-obese populations, leaving a critical evidence gap regarding high-risk patients (1, 10). Moreover, the cost-effectiveness of robotic surgery remains controversial, especially in resource-limited settings.

Therefore, the objective of this study is to retrospectively compare the perioperative outcomes and complication profiles of DV-RH, TLH, and LH in patients with complex large uteri, aiming to provide evidence-based recommendations to support individualized surgical decision-making in clinical practice.

## 2 Materials and methods

### 2.1 Research subjects

**Figure 1:**
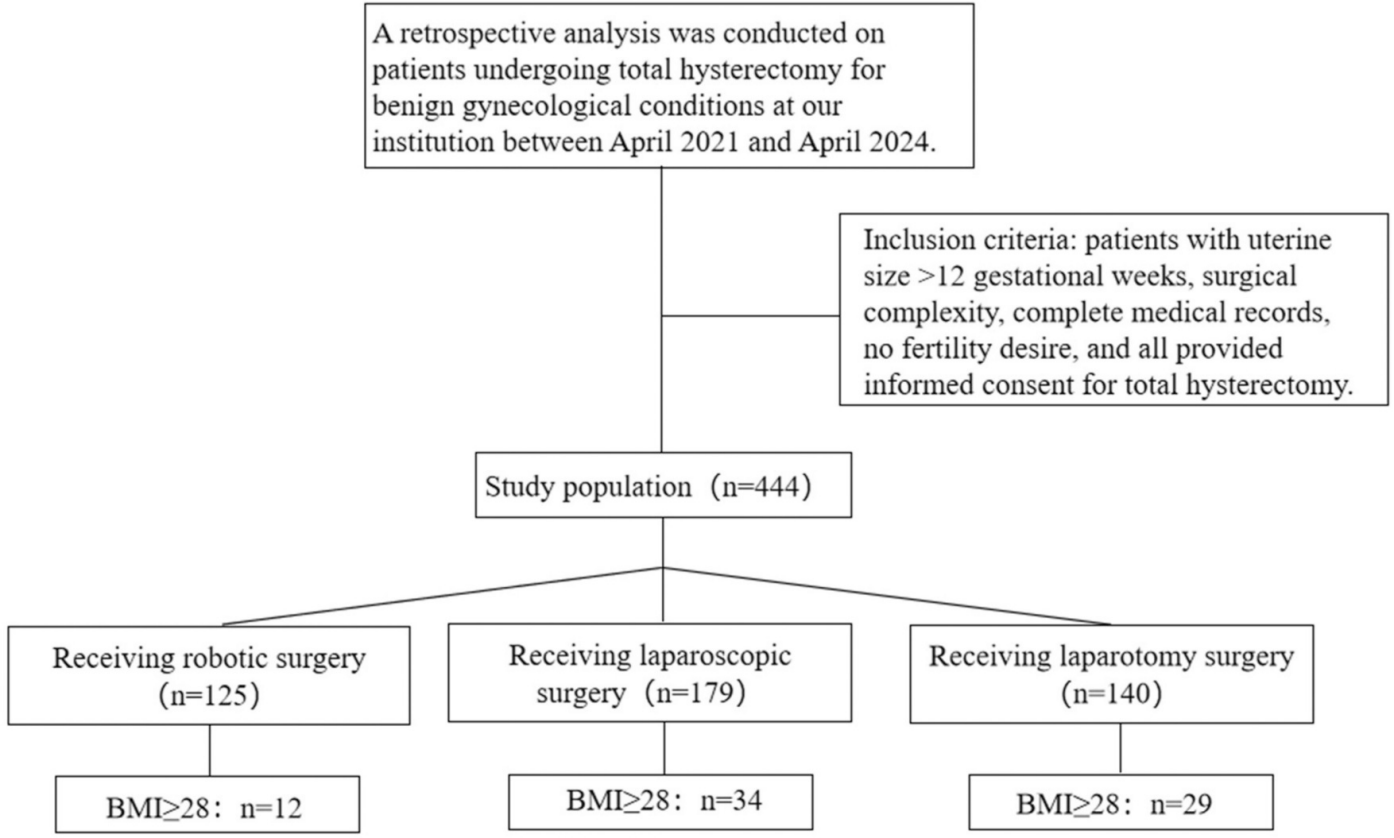
Inclusion flowchart of all enrolled patients.

A retrospective analysis was performed on the clinical data of 444 patients who visited the Department of Gynecology, Ningxia Medical University General Hospital from April 2021 to April 2024 and underwent LH, TLH, or DV-RH due to benign gynecological diseases. In this study, a complex large uterus was defined as one assessed by two senior attending physicians during gynecological examination to be equivalent to ≥12 gestational weeks in size (fundal height above the symphysis pubis by 2–3 fingerbreadths), and also meeting at least one of the following criteria: abnormal uterine morphology or a history of previous abdominopelvic surgery. All patients undergoing hysterectomy for benign conditions between April 2021 and April 2024 were retrospectively reviewed. The surgical approach—total laparoscopic hysterectomy (TLH), open abdominal hysterectomy (LH), or robot-assisted laparoscopic hysterectomy (DV-RH)—was determined after comprehensive counseling and shared decision-making with the patient, taking into account surgical feasibility, individual patient characteristics, and economic considerations (revised to: Allocation to surgical approach was non-randomized and based on shared decision-making, considering: (1) surgical feasibility (e.g., minimally invasive preferred for larger uteri to reduce blood loss); (2) patient factors (e.g., obesity or comorbidities); (3) prior surgical history; and (4) economic accessibility. While this reflects clinical practice, it may contribute to selection bias, as discussed in the limitations.).According to the surgical procedure, they were divided into the LH group (140 cases), the TLH group (179 cases), and the DV-RH group (125 cases) (Figure 1). Patients with a voluminous uterus—typically defined as equivalent to ≥12 weeks of gestation in size—and abnormal uterine morphology are classified as having a complex large uterus; Patients with a BMI ≥28 were classified as obese. Inclusion criteria: Clinically diagnosed (combined with imaging examinations, gynecological examinations, etc.) as requiring hysterectomy due to benign diseases with a complex large uterus, defined as the fundus height at 2–3 transverse fingers above the pubic symphysis (uterine size >12 gestational weeks), and the surgery is complex (such as combined with pelvic adhesions, abnormal uterine position, accompanied by other underlying diseases that increase the difficulty of surgery, etc.); the patient's medical record data is complete; no fertility requirement; signed the informed consent form. Exclusion criteria: Patients with surgical contraindications who cannot tolerate surgery, conversion to laparotomy surgery, preoperative histologic evidence of malignancy or intraoperative findings requiring oncologic staging, and severely missing data that significantly affect the research analysis. This study has been approved by the Ethics Committee of the Ningxia Medical University General Hospital Medical Research Ethics Review Committee on January 24, 2025 (Ethics Approval Number: KYLL-2025–0069).

### 2.2 Surgical instruments and equipment

**Figure 2:**
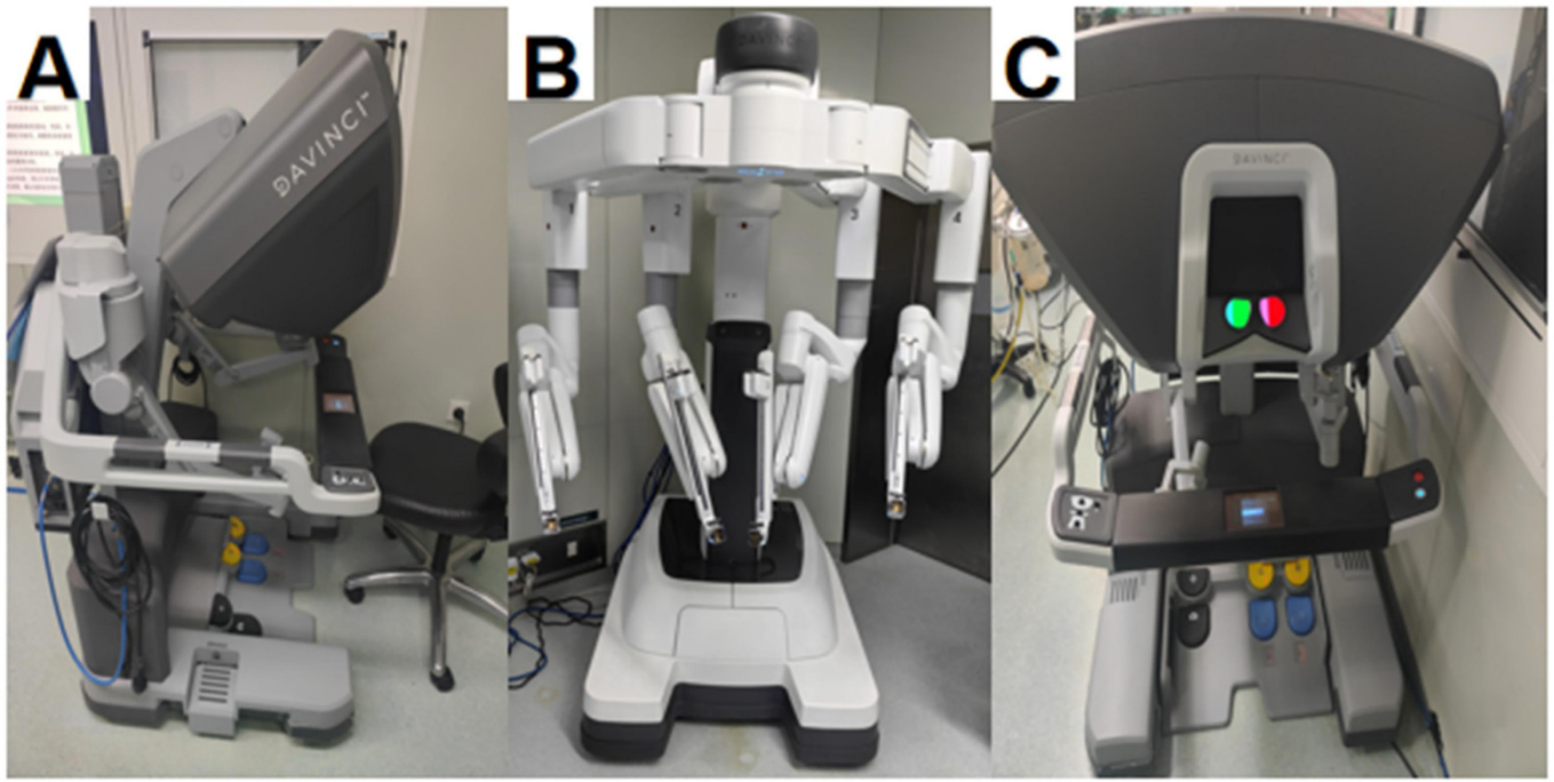
**(A)** External view of the left-side Da Vinci surgical system console; **(B)** the robotic arm component of the intermediate Da Vinci surgical system; **(C)** another perspective of the right-sided Da Vinci surgical system console.

The LH group was prepared with a conventional laparotomy surgical instrument kit, including various surgical knives, tissue scissors, hemostats, etc. The TLH group was equipped with a high-definition laparoscopic system (including a laparoscopic lens, a cold light source, a pneumoperitoneum machine, etc.), and laparoscopic special operating instruments (such as an ultrasonic scalpel, bipolar electrocoagulation forceps, etc.). The DV-RH group was equipped with a Da Vinci robot host, a robotic arm system, and an imaging system (Figure 2).

### 2.3 Surgical methods

#### 2.3.1 Preoperative evaluation

Preoperatively, the patient's current medical history and past medical history were completed, and a comprehensive gynecological examination, relevant laboratory tests, and imaging examinations were performed to formulate an appropriate surgical plan. Preoperative assessment included detailed history-taking, gynecological examination, laboratory tests, and imaging (transvaginal ultrasound or MRI). Endometrial sampling with histologic confirmation was performed in patients with abnormal bleeding or suspicious imaging to exclude malignancy, ensuring all included cases were benign. The patient was instructed to fast and refrain from drinking water 8–12 h before surgery, and skin preparation and blood preparation were carried out. If necessary, a cleansing enema was given twice. The patient was required to sign a surgical consent form (the DV-RH group signed the surgical consent form with a third-party witness). In addition, 1.5 g of cefuroxime sodium for injection was intravenously dripped 30 min before surgery to prevent infection.

#### 2.3.2 Surgical procedures

Procedures were performed by a consistent surgical team led by experienced gynecologists (>10 years in all techniques), with standardized protocols to minimize variability. DV-RH cases were led or supervised by robotic-certified surgeons. This differs from single-surgeon designs, such as Karagün et al. (11), but enhances generalizability.

#### 2.3.3 DV-RH group

**Figure 3:**
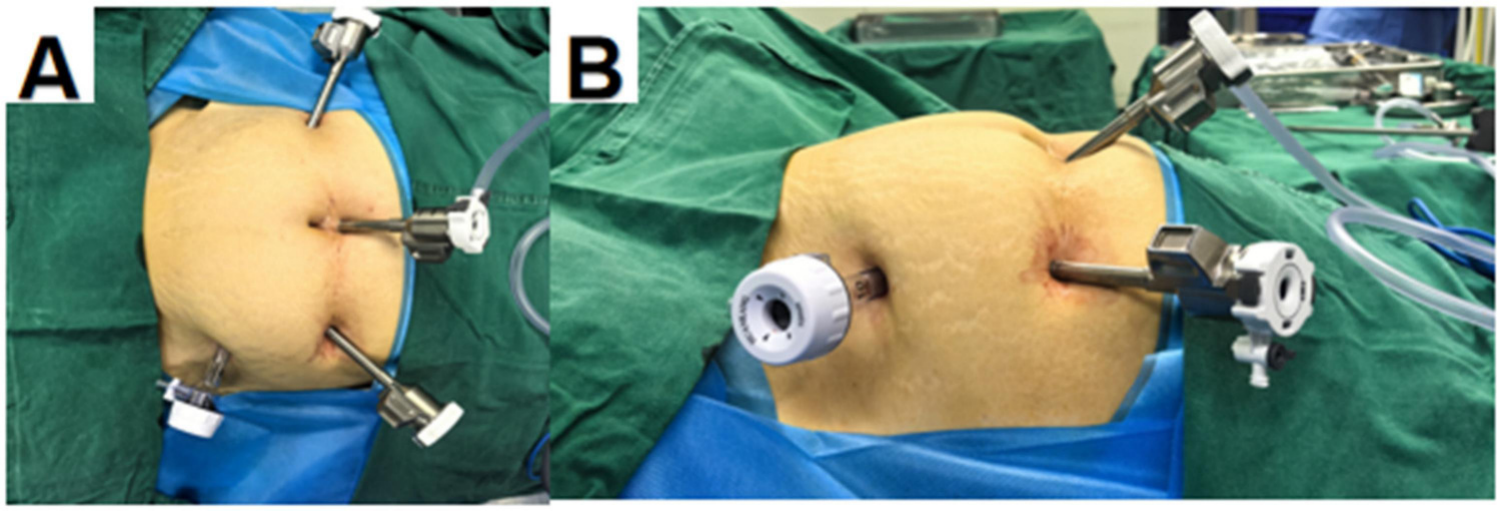
**(A)** Troca layout; **(B)** side view of the troca layout.

**Figure 4:**
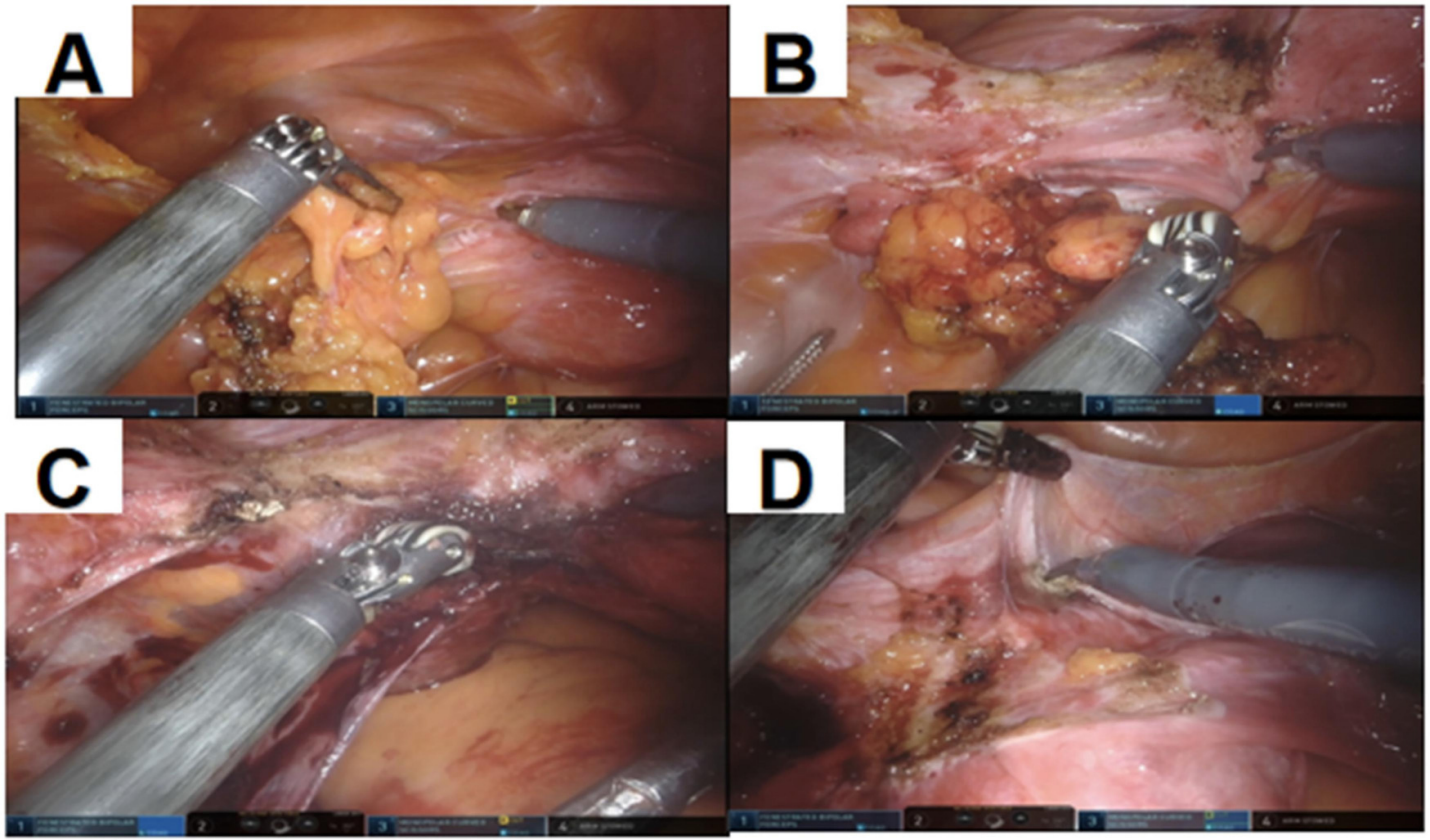
**(A)** Separation of adhesions; **(B)** separation of adhesions; **(C)** skeletonization and transection of uterine vessels; **(D)** open the vesicouterine peritoneal reflection.

All procedures were performed utilizing three robotic arms. The endoscopic arm was equipped with a three-dimensional camera, while robotic arm 1 was fitted with a monopolar electrocautery scissors featuring wristed functionality, and robotic arm 2 was installed with a bipolar forceps. After the induction of anesthesia, the patient was placed in the lithotomy position. Routine disinfection and draping were performed, followed by urinary catheterization and insertion of a uterine manipulator. A 12-mm trocar was placed 2 cm above the umbilicus for the introduction of the laparoscope. Under laparoscopic visualization, two ancillary ports were established bilaterally at the umbilical level, approximately 8 cm from the camera port, and trocars were inserted for the docking of the robotic arms. An additional 10-mm trocar was placed at the left midclavicular line, approximately one-third of the distance between the anterior superior iliac spine and the umbilicus, to serve as an assistant port. Following adequate exposure of the surgical field, a thorough exploration of the pelvic cavity was conducted. The round ligament, fallopian tube, and utero-ovarian ligament (or infundibulopelvic ligament, depending on the patient's age and desire for adnexal preservation) were sequentially transected. Vessels were coagulated and divided using appropriate energy devices, and tissues were dissected and ligated as necessary. The uterus was resected in a stepwise manner and extracted either vaginally or through a mini-laparotomy. Placement of a pelvic drainage tube was determined based on the intraoperative findings, and the abdominal incision was closed in layers (Figures 3, 4).

#### 2.3.4 TLH group

Following the induction of anesthesia, the patient was placed in the dorsal lithotomy position. Standard antiseptic preparation and draping were carried out, followed by urinary catheterization and placement of a uterine manipulator. A 12-mm trocar was inserted 1 cm above the umbilicus for laparoscopic access. Under laparoscopic guidance, two 5-mm ancillary trocars were placed at the bilateral midclavicular lines approximately one-third of the distance from the anterior superior iliac spine to the umbilicus. An additional disposable 5-mm trocar was introduced under endoscopic vision in an avascular area at the left midclavicular line, approximately two-thirds of the distance between the umbilicus and the anterior superior iliac spine. The subsequent surgical steps, including dissection, resection, and specimen extraction, were consistent with those described in the RH group.

#### 2.3.5 LH group

All procedures were performed via conventional open laparotomy. The patient was placed in the supine position. After successful anesthesia induction, the surgical field was sterilized (extending from below the xiphoid process to the upper third of the thighs and laterally to the midaxillary lines) and draped under aseptic conditions. A Foley catheter was inserted. A longitudinal midline abdominal incision approximately 10–15 cm in length was made, and the abdominal layers were dissected sequentially to access the peritoneal cavity. A comprehensive exploration of the abdominopelvic cavity was performed. Retractors were placed to adequately expose the uterus and adnexal structures. The round ligament, fallopian tube, and utero-ovarian ligament (or infundibulopelvic ligament, depending on the patient's age and desire for adnexal preservation) were sequentially transected. Vascular control was achieved using electrocautery, ligation, and sharp dissection. The uterus was resected in a stepwise fashion and removed *en bloc*. The vaginal cuff was disinfected. Placement of a pelvic drain was determined according to intraoperative conditions. The vaginal cuff was sutured, and the abdominal incision was closed in layers after confirming that all surgical instruments and sponges were accounted for.

Crucially, for all three surgical approaches, no local anesthetic agents were infiltrated at the incision sites prior to skin incision. Furthermore, in strict adherence to the principles of oncological surgery (tumor-free technique), a mechanical morcellator was intentionally not used in any of the procedures across all groups to avoid the potential risk of tissue dissemination.

### 2.4 Observation indicators

General information: Age, body mass index (BMI), gravidity, parity, preoperative hemoglobin (Hb), uterine size, comorbidities, history of abdominal surgery, main diagnosis of hysterectomy, etc.; Perioperative related indicators: Postoperative hemoglobin (Hb), operation time, blood loss, postoperative 12-h visual analogue scale (VAS) score, gastrointestinal function recovery time, postoperative hospitalization time, indwelling time of urinary catheter and drainage tube, postoperative antibiotic escalation rate, use of postoperative analgesic drugs, etc.; Occurrence of complications: Urinary system injury, intestinal injury, vascular injury, intestinal obstruction, incisional hernia, incision infection, pelvic infection, deep vein thrombosis of the lower extremities, poor healing of the vaginal stump, abdominal wall hematoma, pulmonary embolism, subcutaneous emphysema, encapsulated effusion, lymphocele retention, etc. The complication grading was based on the Clavien-Dindo grading standard (9).

### 2.5 Statistical methods

All statistical analyses were performed using SPSS 26.0 statistical software. Normally distributed measurement data were expressed as mean ± standard deviation (±s), and one-way analysis of variance was used for difference comparison. Non-normally distributed measurement data were expressed as median (M) and quartiles (P25, P75), and non-parametric tests were used for comparison. Counting data were expressed as number of cases) [*n* (%)], and the chi-square test was used. *P* < 0.05 was considered statistically significant.

## 3 Results

### 3.1 Baseline characteristics

There were no statistically significant differences among the three groups in terms of age, BMI, gravidity, preoperative hemoglobin levels, comorbidities, or history of abdominal surgery. However, parity, uterine size, and the primary indication for hysterectomy differed significantly among the groups (*P* < 0.05) as detailed data shown in Table 1. Nine cases (5.0%) in the TLH group required conversion to open surgery due to adhesions or poor visualization, with none in DV-RH.

**Table 1:** Comparison of general information of the three groups of patients.

Indicator name	DV-RH Group (*n* = 125)	TLH Group (*n* = 179)	LH Group (*n* = 140)	Statistical Value	*P*-Value
Age	46 (49, 52)	47 (50, 52)	47 (49, 51)	0.923	0.337
BMI	22.31 (24.03, 26.56)	22.59 (24.61, 27.06)	22.88 (24.76, 27.43)	2.03	0.154
Gravidity	2 (3, 4)	2 (3, 4)	2 (3, 4)	0.009	0.925
Parity	1 (1, 2)	1 (2, 2)	1 (1, 2)	4.779	**0**.**029**
Preoperative Hemoglobin	92 (115, 135)	96.5 (121, 136)	89.5 (110.5, 130)	1.407	0.236
Comorbidities^a^				4.515	0.105
No	58 (46.4%)	93 (52%)	56 (40%)		
Yes	67 (53.6%)	86 (48%)	84 (60%)		
Uterine Size				91.625	**<0**.**001**
12–16 Weeks	56 (44.8%)	127 (70.9%)	24 (17.1%)		
≥ 16 Weeks	69 (55.2%)	52 (29.1%)	116 (82.9%)		
History of Abdominal Surgery				5.265	0.072
No	56 (44.8%)	102 (57%)	66 (47.1%)		
Yes	69 (55.2%)	77 (43%)	74 (52.9%)		
Main Diagnosis of Hysterectomy				11.338	**0**.**023**
Uterine Leiomyoma	75 (60%)	122 (68.2%)	107 (76.4%)		
Adenomyoma	50 (40%)	54 (30.2%)	32 (22.9%)		
Abnormal Uterine Bleeding	0 (0%)	3 (1.7%)	1 (0.7%)		

^a^ Comorbidities include hypertension, diabetes, coronary atherosclerotic heart disease, anemia.

*P* < 0.05 was considered to be statistically significant.

Bold values indicate statistically significant differences (*P* < 0.05) compared with the other two surgical groups.

### 3.2 Comparison of perioperative indicators in the three patient groups

As shown in Table 2, the hospitalization cost in the DV-RH group was significantly higher than in both the TLH and LH groups (*P* < 0.001; *P* < 0.001), while no significant difference was observed between the TLH and LH groups (*P* = 0.258). Intraoperative blood loss was significantly lower in the DV-RH group compared to the LH group (*P* < 0.001), and also significantly lower in the TLH group compared to the LH group (*P* < 0.001); however, there was no significant difference between the DV-RH and TLH groups (*P* = 0.241). Postoperative VAS scores were significantly lower in the DV-RH group than in the TLH and LH groups (*P* < 0.001; *P* < 0.001), and the TLH group showed significantly lower scores than the LH group (*P* = 0.022). Gastrointestinal function recovery time was significantly shorter in the DV-RH group than in the LH group (*P* < 0.001), and also shorter in the TLH group than in the LH group (*P* = 0.002). There was no significant difference between the DV-RH and TLH groups (*P* = 0.062). Postoperative hospitalization duration was shorter in the DV-RH group compared to the LH group (*P* < 0.001), and also shorter in the TLH group than in the LH group (*P* < 0.001). No significant difference was observed between the DV-RH and TLH groups (*P* = 0.901). The DV-RH group had a shorter drainage duration than the LH group (*P* = 0.015), and the TLH group was shorter than the LH group (*P* < 0.001). No significant differences were found between the DV-RH and TLH groups (*P* = 0.755; *P* = 0.183). The incidence of postoperative analgesic use was significantly lower in the DV-RH and TLH groups than in the LH group (*P* = 0.022; *P* = 0.018), with no significant difference between the DV-RH and TLH groups (*P* = 0.869). The use of postoperative antibiotics was significantly lower in the DV-RH group compared to both the TLH and LH groups (*P* < 0.001; *P* < 0.001), and no significant difference was observed between the TLH and LH groups (*P* = 0.124). No significant differences were found among the three groups in terms of operation duration, drainage days, or 24-h postoperative drainage volume (*P* > 0.05).

**Table 2:** Comparison of perioperative-related indicators in the three groups.

Indicator name	DV-RH Group (*n* = 125)	TLH Group (*n* = 179)	LH Group (*n* = 140)	Statistical Value	*P*-Value
Hospitalization cost (RMB)	40,878.05 (39,749.66, 42,325.50)	12,176.11 (10,846.45, 13,627.43)	12,305.35 (11,185.95, 14,178.96)	259.255	**<0**.**001**
Procedure duration (min)	75 (92, 121)	65 (90, 110.5)	60 (90, 110)	5.87	0.053
Amount of bleeding (ml)	20 (30, 50)	20 (30, 50)	50 (50, 100)	56.215	**<0**.**001**
Postoperative VAS score	2 (2, 3)	3 (3, 3)	3 (3, 3)	85.607	**<0**.**001**
Time to recovery of gastrointestinal function (d)	2 (2, 2)	2 (2, 2)	2 (2, 2)	29.664	**<0**.**001**
Number of days in hospital after surgery	2 (2, 3)	2 (3, 3)	3 (3, 4)	25.229	**<0**.**001**
Days of days of the drain	0 (0, 0)	0 (0, 0)	0 (0, 0)	4.357	0.113
Number of days of urinary tract lien	2 (2, 3)	2 (3, 3)	2 (3, 3)	8.115	**0**.**017**
Flow rate was induced at 24 h after surgery	0 (0, 0)	0 (0, 0)	0 (0, 0)	5.615	0.06
Intraoperative blood transfusion				0.932	0.628
No	114 (91.2%)	157 (87.7%)	125 (89.3%)		
Yes	11 (8.8%)	22 (12.3%)	15 (10.7%)		
Antibiotics were escalated after surgery				7.675	**0**.**022**
No	107 (85.6%)	152 (84.9%)	104 (74.3%)		
Yes	18 (14.4%)	27 (15.1%)	36 (25.7%)		
Postoperative use of analgesic drugs				110.253	**<0**.**001**
No	46 (36.8%)	5 (2.8%)	0 (0%)		
Yes	79 (63.2%)	174 (97.2%)	140 (100%)		

Bold values indicate statistically significant differences (*P* < 0.05) compared with the other two surgical groups.

### 3.3 Comparison of surgical complications

Among all surgical complications, only incision infection showed a statistically significant difference among the three groups (*P* < 0.05), while other complications such as urinary tract injury and bowel injury were not significantly different between groups (*P* > 0.05), See Table 3.

**Table 3:** Comparison of surgical complications among the three patient groups.

Indicator name	DV-RH Group (*n* = 125)	TLH Group (*n* = 179)	LH Group (*n* = 140)	Statistical Value	*P*-Value
Urinary system injury	1 (0.8%)	6 (3.4%)	1 (0.7%)	4.297	0.117
Intestinal canal injury	0 (0%)	1 (0.6%)	0 (0%)	1.484	0.476
Injury of blood vessel	0 (0%)	0 (0%)	0 (0%)		
Intestinal obstruction	0 (0%)	1 (0.6%)	2 (1.4%)	2.069	0.355
Incisional hernia	0 (0%)	0 (0%)	0 (0%)		
Infection of incisional wound	0 (0%)	0 (0%)	3 (2.1%)	6.559	**0**.**038**
Pelvic infection	1 (0.8%)	3 (1.7%)	0 (0%)	2.491	0.288
Deep vein thrombosis in both lower limbs	0 (0%)	1 (0.6%)	0 (0%)	1.484	0.476
Poor healing of the vaginal stump	0 (0%)	0 (0%)	0 (0%)		
Abdominal wall hematoma	0 (0%)	0 (0%)	0 (0%)		
Pulmonary embolism	0 (0%)	2 (1.1%)	0 (0%)	2.974	0.226
Subcutaneous emphysema	1 (0.8%)	0 (0%)	0 (0%)		
Encapsulated effusion	0 (0%)	0 (0%)	0 (0%)		
Lymphocele retention	0(0%)	0(0%)	0(0%)		

Bold values indicate statistically significant differences (*P* < 0.05) compared with the other two surgical groups.

### 3.4 Comparison of the severity of surgical complications in the three patient groups

The incidence of grade II and III complications in the TLH group was higher than that in the DV-RH and LH groups. Especially for grade III complications, the incidence in the TLH group reached 3.4%. The incidence of various grades of complications in the DV-RH and LH groups was lower, and most of them occurred in grades I and II. In terms of grade IV complications, 2 cases occurred in the TLH group, and no grade IV complications occurred in the other two groups (Table 4).

**Table 4:** Comparison of surgical complications of the three groups of patients according to Clavein-Dindo classification [*n* (%)].

Indicator name	DV-RH Group (*n* = 125)	TLH Group (*n* = 179)	LH Group (*n* = 140)	Statistical Value	*P*-Value
Grade I	1 (0.8%)	0 (0%)	3 (2.1%)	4.06	0.131
Grade II	1 (0.8%)	6 (3.4%)	1 (1.4%)	2.782	0.49
Grade III	1 (0.8%)	6 (3.4%)	1 (0.7%)	4.076	0.131
Grade IV	0 (0%)	2 (1.1%)	0 (0%)	2.974	0.226

### 3.5 Comparison of the indicators in the population of enlarged uterus

Comparison of perioperative related indicators: The DV-RH group exhibited significantly higher hospitalization costs than the TLH and LH groups (both *P* < 0.001), while no difference was observed between TLH and LH (*P* = 0.944). Intraoperative blood loss was lower in DV-RH and TLH compared to LH (both *P* < 0.010), with no intergroup difference between DV-RH and TLH (*P* = 0.921). Postoperative VAS scores were lowest in DV-RH (vs. TLH/LH, both *P* < 0.001), followed by TLH (vs. LH, *P* = 0.019). Gastrointestinal recovery was faster in DV-RH and TLH than LH (both *P* < 0.05), with comparable outcomes between DV-RH and TLH (*P* = 0.411). DV-RH had shorter hospital stays than LH (*P* = 0.014), but no differences existed among other comparisons. TLH required fewer days with drainage tubes than DV-RH/LH (both *P* < 0.05), while DV-RH showed lower 24-h drainage volume than TLH (*P* = 0.008). Postoperative analgesic use was reduced in DV-RH (vs. TLH/LH, both *P* < 0.001) and TLH (vs. LH, *P* = 0.009). No significant differences were noted in operative time, urinary catheter duration, intraoperative transfusion, or antibiotic escalation (both *P* > 0.05). See Table 5.

**Table 5:** Comparison of perioperative indicators in the population with enlarged uterus.

Indicator name	DV-RH Group (*n* = 69)	TLH Group (*n* = 52)	LH Group (*n* = 116)	Statistical Value	*P*-Value
Hospitalization cost (RMB)	40,937.775 (39,811.93,42, 325.4975)	12,261.945 (10,833.8725, 14,237.325)	12,210.6 (11,102.2925, 13,900.0475)	140.291	**<0**.**001**
Procedure duration (min)	75 (92, 130)	70.5 (90, 120)	61 (90, 111.5)	2.161	0.339
amount of bleeding (mL)	30 (50, 50)	20 (50, 50)	50 (50, 100)	14.327	**0**.**001**
Postoperative VAS score	2 (2, 3)	2 (3, 3)	3 (3, 3)	67.476	**<0**.**001**
Time to recovery of gastrointestinal function (d)	2 (2, 2)	2 (2, 2)	2 (2, 2)	14.506	**0**.**001**
Number of days in hospital after surgery	2 (3, 4)	2 (3, 4)	3 (3, 4)	6.256	**0**.**044**
Days of days of the drain	0 (0, 0)	0 (0, 0)	0 (0, 0)	7.812	**0**.**02**
Number of days of urinary tract lien	2 (2, 3)	2 (3, 3)	2 (3, 3)	1.929	0.381
Flow rate was induced at 24 h after surgery	0 (0, 0)	0 (0, 12.5)	0 (0, 0)	10.471	**0**.**005**
Intraoperative blood transfusion				0.16	0.923
No	62 (89.9%)	46 (88.5%)	102 (87.9%)		
Yes	7 (10.1%)	6 (11.5%)	14 (12.1%)		
Antibiotics were escalated after surgery				51.064	**<0**.**001**
No	23 (33.3%)	3 (5.8%)	0 (0%)		
Yes	46 (66.7%)	49 (94.2%)	116 (100%)		
Postoperative use of analgesic drugs				2.664	0.264
No	57 (82.6%)	44 (84.6%)	87 (75%)		
Yes	12 (17.4%)	8 (15.4%)	29 (25%)		

Bold values indicate statistically significant differences (*P* < 0.05) compared with the other two surgical groups.

Comparison of Surgical Complications Among Different Groups of Patients with Giant Uterus: There was no statistically significant difference in surgical complications among the three groups of patients (*P* > 0.05). See Table 6.

**Table 6:** Comparison of surgical complications among patients in each group with enlarged uterus.

Indicator name	DV-RH Group (*n* = 69)	TLH Group (*n* = 52)	LH Group (*n* = 116)	Statistical Value	*P*-Value
Urinary system injury	1 (1.4%)	1 (1.9%)	1 (0.9%)	0.35	0.84
Intestinal canal injury	0 (0%)	1 (1.9%)	0 (0%)	3.573	0.168
Injury of blood vessel	0 (0%)	0 (0%)	0 (0%)		
Intestinal obstruction	0 (0%)	1 (1.9%)	1 (0.9%)	1.311	0.519
Incisional hernia	0 (0%)	0 (0%)	0 (0%)		
Infection of incisional wound	0 (0%)	0 (0%)	3 (2.6%)	3.169	0.205
Pelvic infection	0 (0%)	1 (1.9%)	0 (0%)	3.573	0.168
Deep vein thrombosis in both lower limbs	0 (0%)	0 (0%)	0 (0%)		
Poor healing of the vaginal stump	0 (0%)	0 (0%)	0 (0%)		
Abdominal wall hematoma	0 (0%)	0 (0%)	0 (0%)		
Pulmonary embolism	0 (0%)	1 (1.9%)	0 (0%)	3.573	0.168
Subcutaneous emphysema	0 (0%)	0 (0%)	0 (0%)		
Encapsulated effusion	0 (0%)	0 (0%)	0 (0%)		
Lymphocele retention	0 (0%)	0 (0%)	0 (0%)		

Comparison of surgical complications among different Clavein-Dindo grades in three groups of patients with enlarged uterus: No grade II complications were observed in the DV-RH group. In contrast, grade II complications occurred in 3 cases (5.8%) in the TLH group and 1 case (0.9%) in the LH group, demonstrating a statistically significant intergroup difference (*P* = 0.032). *Post hoc* analysis revealed that the TLH group had a significantly higher incidence of grade II complications compared to both the DV-RH and LH groups. However, no significant differences were observed among the three groups in the incidence of grade I, III, or IV complications (*P* > 0.05; Table 7).

**Table 7:** Comparison of surgical complications at different Clavein-Dindo grades in the super-large uterus population among the three groups [cases (%)].

Indicator name	DV-RH Group (*n* = 69)	TLH Group (*n* = 52)	LH Group (*n* = 116)	Statistical Value	*P*-Value
Grade I	0 (0%)	0 (0%)	3 (2.6%)	1.481	0.477
Grade II	0 (0%)	3 (5.8%)	1 (0.9%)	6.882	**0**.**032**
Grade III	1 (1.4%)	1 (1.9%)	1 (0.9%)	0.35	0.84
Grade IV	0 (0%)	1 (1.9%)	0 (0%)	3.573	0.168

Bold values indicate statistically significant differences (*P* < 0.05) compared with the other two surgical groups.

### 3.6 Comparison of indicators in the population of obese patients

Comparison of perioperative related indicators: Significant differences were observed among the DX-RH, TLH, and LH groups in several perioperative parameters (Table 8). The DX-RH group incurred substantially higher hospitalization costs compared to the TLH and LH groups (*P* < 0.001). Intraoperative blood loss was significantly lower in the DX-RH and TLH groups than in the LH group (*P* < 0.001). The LH group also demonstrated higher postoperative pain scores compared to the DX-RH group (*P* = 0.004).Notably, the DX-RH group had a lower postoperative analgesic requirement (*P* < 0.001) and a reduced need for escalated antibiotics (*P* = 0.044). However, no significant differences were found in procedure duration (*P* = 0.132), gastrointestinal recovery time (*P* = 0.354), postoperative hospital stay (*P* = 0.427), or urinary catheter duration (*P* = 0.925). Drain use and 24-hour postoperative flow rates differed marginally among groups (*P* = 0.029 and *P* = 0.016, respectively).

**Table 8:** Comparative analysis of perioperative outcomes in obese patients.

Indicator name	DV-RH Group (*n* = 12)	TLH Group (*n* = 34)	LH Group (*n* = 29)	Statistical Value	*P*-Value
Hospitalization cost (RMB)	41,342.58 (39,954.335, 44,247.8875)	12,632.92 (11,284.0375, 14,819.9875)	11,970.17 (11,375.555, 14,157.625)	30.249	**<0** **.** **001**
Procedure duration (min)	85 (94, 124.5)	66 (80.5, 112)	83 (90, 90)	4.057	0.132
Amount of bleeding (mL)	20 (30, 40)	20 (25, 50)	50 (50, 100)	21.381	**<0**.**001**
Postoperative VAS score	2 (2.5, 3)	3 (3, 3)	3 (3, 3)	10.942	**0**.**004**
Time to recovery of gastrointestinal function (d)	2 (2, 2)	2 (2, 2)	2 (2, 2)	2.076	0.354
Number of days in hospital after surgery	2 (3, 3.5)	2 (3, 4)	3 (3, 4)	1.703	0.427
Days of days of the drain	0 (0, 0)	0 (0, 0)	0 (0, 0)	7.064	**0**.**029**
Number of days of urinary tract lien	2 (3, 3)	2 (3, 3)	2 (3, 3)	0.155	0.925
Flow rate was induced at 24 h after surgery	0 (0, 0)	0 (0, 0)	0 (0, 0)	8.269	**0**.**016**
Intraoperative blood transfusion				1.083	0.582
No	11 (91.7%)	29 (85.3%)	27 (93.1%)		
Yes	1 (8.3%)	5 (14.7%)	2 (6.9%)		
Antibiotics were escalated after surgery				6.264	**0**.**044**
No	11 (91.7%)	30 (88.2%)	19 (65.5%)		
Yes	1 (8.3%)	4 (11.8%)	10 (34.5%)		
Postoperative use of analgesic drugs				22.183	**<0**.**001**
No	4 (33.3%)	0 (0%)	0 (0%)		
Yes	8 (66.7%)	34 (100%)	29 (100%)		

Bold values indicate statistically significant differences (*P* < 0.05) compared with the other two surgical groups.

Comparison of Surgical Complications Among Different Groups in the obese patients: As shown in Table 9, urinary system injury occurred in 2 (5.9%) patients in the TLH group but was absent in the DV-RH and LH groups (*P* = 0.290). Intestinal obstruction and pulmonary embolism were observed in 1 (2.9%) TLH case each, while pelvic infection occurred in 1 (8.3%) DV-RH and 1 (2.9%) TLH patient (both *P* > 0.05). No significant differences were found in other complications (intestinal/vascular injury, incisional hernia, wound infection, DVT, vaginal stump healing, hematoma, subcutaneous emphysema, effusion, or lymphocele), with incidences of 0% across groups (both *P* > 0.05).

**Table 9:** Comparison of surgical complications among different groups in the obese population.

Indicator name	DV-RH Grou (*n* = 12)	TLH Group (*n* = 34)	LH Group (*n* = 29)	Statistical Value	*P*-Value
Urinary system injury	0 (0%)	2 (5.9%)	0 (0%)	2.478	0.29
Intestinal canal injury	0 (0%)	0 (0%)	0 (0%)		
Injury of blood vessel	0 (0%)	0 (0%)	0 (0%)		
Intestinal obstruction	0 (0%)	1 (2.9%)	0 (0%)	1.222	0.543
Incisional hernia	0 (0%)	0 (0%)	0 (0%)		
Infection of incisional wound	0 (0%)	0 (0%)	0 (0%)		
Pelvic infection	1 (8.3%)	1 (2.9%)	0 (0%)	2.289	0.318
Deep vein thrombosis in both lower limbs	0 (0%)	0 (0%)	0 (0%)		
Poor healing of the vaginal stump	0 (0%)	0 (0%)	0 (0%)		
Abdominal wall hematoma	0 (0%)	0 (0%)	0 (0%)		
Pulmonary embolism	0 (0%)	1 (2.9%)	0 (0%)	1.222	0.543
Subcutaneous emphysema	0 (0%)	0 (0%)	0 (0%)		
Encapsulated effusion	0 (0%)	0 (0%)	0 (0%)		
Lymphocele retention	0 (0%)	0 (0%)	0 (0%)		

Comparison of Surgical Complications by Clavien-Dindo Classification Among Different Groups in the obese patients: The incidence of postoperative complications stratified by Clavien-Dindo classification revealed no statistically significant differences among the DV-RH, TLH, and LH groups in the obese population (Table 10). Grade II complications occurred in 1 patient (8.3%) in the DV-RH group and 2 patients (5.9%) in the TLH group, with none reported in the LH group (*P* = 0.348). Grade III complications were observed exclusively in the TLH group (*P* = 0.290). A single Grade IV complication(2.9%) was recorded in the TLH group, with no occurrences in the other groups (*P* = 0.543). No Grade I complications were reported in any cohort.

**Table 10:** Different groups in the obese population.

Indicator name	DV-RH Group (*n* = 12)	TLH Group (*n* = 34)	LH Group (*n* = 29)	Statistical Value	*P*-Value
Grade I	0 (0%)	0 (0%)	0 (0%)		
Grade II	1 (8.3%)	2 (5.9%)	0 (0%)	2.109	0.348
Grade III	0 (0%)	2 (5.9%)	0 (0%)	2.478	0.29
Grade IV	0 (0%)	1 (2.9%)	0 (0%)	1.222	0.543

## 4 Discussion

Although the General Hospital of Ningxia Medical University introduced the Da Vinci Surgical System in April 2021, marking a new phase of development for minimally invasive surgery in the region, clinical data regarding robot-assisted laparoscopic procedures in Ningxia remain relatively scarce. Existing literature predominantly focuses on large medical centers in more developed eastern regions of China, which may not adequately reflect the practical applicability and effectiveness of this advanced technology in western China. Therefore, this study systematically analyzes clinical application data of robotic surgery at Ningxia Medical University General Hospital, aiming to evaluate its safety, efficacy, and short-term outcomes. The findings are expected to provide evidence-based support for the standardized application and localized development of this high-end minimally invasive technology in the region, thereby facilitating the optimization of regional healthcare resources and the advancement of surgical techniques. This retrospective study systematically compared the perioperative outcomes and complication profiles of three commonly used surgical approaches—DV-RH, TLH, and LH—in patients with complex large uteri, including a substantial proportion with prior abdominal surgeries and presumed obesity. Our findings demonstrate that DV-RH was associated with the lowest intraoperative blood loss, shorter gastrointestinal recovery time, lower postoperative pain, and fewer postoperative complications, despite being more frequently selected for technically demanding cases involving uterine volumes ≥16 gestational weeks. DV-RH showed advantages in blood loss and pain, though differences with TLH were not always statistically significant, suggesting comparable efficacy in select cases. Notably, although DV-RH incurred the highest hospitalization cost, it showed a clear advantage in surgical precision and postoperative recovery. These results underscore the clinical utility of robotic-assisted surgery in managing high-risk gynecologic patients, particularly when both uterine size and abdominal surgical history increase procedural complexity.

Consistent with previous studies, our analysis confirms the superiority of minimally invasive approaches over open surgery in reducing surgical trauma and accelerating recovery. Both the DV-RH and TLH groups had significantly less blood loss compared to the LH group, aligning with reports that laparoscopic and robotic techniques are associated with decreased vascular injury due to enhanced visualization and finer instrument control (12). However, unlike prior literature that suggests a significant advantage of DV-RH over TLH in blood conservation (10, 13, 14), we did not observe a statistically significant difference between the two in this regard. This discrepancy may be explained by the fact that the DV-RH group in our study included more patients with extremely large uteri (≥16 weeks) and prior abdominal surgeries, likely increasing surgical difficulty and counterbalancing the blood-sparing benefits of robotic precision.

Perioperative advantages of DV-RH may be partly attributable to surgeon experience and selection criteria. For instance, allocation biases (e.g., larger uteri or higher BMI in DV-RH) could influence outcomes, as could varying expertise levels. While our team standardization mitigated this, unadjusted factors like intraoperative uterine weight may persist, warranting caution in generalization.

Another important finding concerns postoperative pain and recovery. The DV-RH group exhibited the lowest postoperative VAS scores and fastest gastrointestinal function recovery, which corroborates evidence that robotic systems allow for more delicate tissue handling and reduced traction on visceral structures (8). Interestingly, although TLH is generally considered an effective minimally invasive option, it showed relatively higher postoperative pain scores and a greater need for analgesics compared to DV-RH. This may reflect limitations in instrument articulation and exposure when dealing with massive uteri, potentially leading to increased manipulation of surrounding tissues and higher rates of conversion to open surgery. In our cohort, 9 TLH cases required conversion, primarily due to poor visualization and dense adhesions, which may have also contributed to higher complication rates and prolonged recovery, aligning with Delphi consensus on standardized workflows Nyangoh Timoh et al. (15).

The Clavien-Dindo analysis of postoperative complications further strengthens the case for DV-RH in high-complexity hysterectomies. Although the overall complication rates were similar across groups, the TLH group had a significantly higher proportion of grade II complications. This observation aligns with findings by Mushtaq et al (16), who reported increased morbidity in laparoscopic hysterectomies for large uteri compared to robotic procedures. This aligns with Karagün et al. (11), who reported fewer complications with minimally invasive approaches for endometrial cancer staging when performed by surgeons with standardized expertise. While LH remains a reliable approach in many institutions, its association with longer recovery, higher infection risk, and more extensive tissue trauma limits its desirability, especially in younger or obese patients with greater sensitivity to postoperative morbidity.

Importantly, although this study did not stratify patients directly by body mass index (BMI), the inclusion of numerous patients with suspected obesity—indicated by clinical records and the presence of comorbidities such as hypertension and diabetes—offers insights into the utility of robotic assistance in this subpopulation. Prior literature consistently highlights the benefits of robotic surgery in obese patients, including lower conversion rates, reduced blood loss, and fewer wound complications (2). The enhanced dexterity and stability of the robotic system may help overcome technical challenges posed by visceral adiposity and limited pelvic access, making DV-RH a rational choice in obese women with large or adherent uteri.

Despite its strengths, this study has several limitations. First, its retrospective design is inherently prone to selection bias, particularly in the assignment of surgical modality, which was influenced by patient preference, uterine size, and surgeon expertise. Although we adjusted for several confounders, unmeasured variables such as surgeon experience and intraoperative uterine weight may still have influenced outcomes. Second, our categorization of uterine size based on gestational weeks, while practical, lacks the objectivity of measured uterine weight or volume. Third, the cost-effectiveness of DV-RH was not assessed in detail beyond hospitalization costs, and future analyses should incorporate long-term outcomes, including time to return to work and quality of life metrics.

Nonetheless, this study provides robust, clinically relevant evidence supporting the use of DV-RH in managing complex hysterectomy cases, especially among women with enlarged uteri and suspected obesity. By directly comparing three surgical techniques in a real-world cohort and stratifying by uterine size, we add new empirical data to the ongoing debate over surgical route selection in gynecology. Our findings emphasize the importance of tailoring surgical approaches to anatomical and metabolic complexities, rather than relying on a one-size-fits-all strategy. Our findings also highlight the need for uniform workflows in robotic hysterectomy, as emphasized in the Delphi-based consensus by Nyangoh Timoh et al. (15), which advocates standardized procedural models to ensure consistency.

In conclusion, DV-RH appears to offer the most favorable balance of safety, precision, and recovery outcomes in complex hysterectomy patients, particularly those with large uteri or prior abdominal surgeries. However, its high cost and limited availability may restrict widespread adoption. Further multicenter, prospective trials with stratified analysis by BMI, uterine volume, and surgical complexity are warranted to refine patient selection criteria, optimize resource allocation, and support evidence-based guidelines for hysterectomy in high-risk populations.

## 5 Conclusion

This study shows that DV-RH offers advantages in reducing blood loss, pain, and complications in complex large uteri. TLH provides comparable outcomes in some cases at a lower cost. DV-RH may be preferred for technically challenging cases, while TLH remains a cost-effective alternative.

## Data Availability

The original contributions presented in the study are included in the article/Supplementary Material, further inquiries can be directed to the corresponding author.
